# Non-random clustering of stress-related genes during evolution of the *S. cerevisiae *genome

**DOI:** 10.1186/1471-2148-6-58

**Published:** 2006-07-21

**Authors:** Debra T Burhans, Lakshmi Ramachandran, Jianxin Wang, Ping Liang, Hugh G Patterton, Michael Breitenbach, William C Burhans

**Affiliations:** 1 Dept. of Computer Science and Bioinformatics Program, Canisius College, Buffalo NY, 14208, USA; 2 Dept. of Cell Stress Biology, Roswell Park Cancer Institute, Buffalo, NY 14263, USA; 3 Dept. of Cancer Genetics, Roswell Park Cancer Institute, Buffalo, NY 14263, USA; 4 Laboratory for Epigenomics and DNA Function, Department of Biotechnology University of the Free State, PO Box 339, Bloemfontein 9300, South Africa; 5 Dept. of Genetics, Salzburg University, Salzburg, Austria

## Abstract

**Background:**

Coordinately regulated genes often physically cluster in eukaryotic genomes, for reasons that remain unclear.

**Results:**

Here we provide evidence that many *S. cerevisiae *genes induced by starvation and other stresses reside in non-random clusters, where transcription of these genes is repressed in the absence of stress. Most genes essential for growth or for rapid, post-transcriptional responses to stress in cycling cells map between these gene clusters. Genes that are transcriptionally induced by stresses include a large fraction of rapidly evolving paralogues of duplicated genes that arose during an ancient whole genome duplication event. Many of these rapidly evolving paralogues have acquired new or more specialized functions that are less essential for growth. The slowly evolving paralogues of these genes are less likely to be transcriptionally repressed in the absence of stress, and are frequently essential for growth or for rapid stress responses that may require constitutive expression of these genes in cycling cells.

**Conclusion:**

Our findings suggest that a fundamental organizing principle during evolution of the *S. cerevisiae *genome has been clustering of starvation and other stress-induced genes in chromosome regions that are transcriptionally repressed in the absence of stress, from which most genes essential for growth or rapid stress responses have been excluded. Chromatin-mediated repression of many stress-induced genes may have evolved since the whole genome duplication in parallel with functions for proteins encoded by these genes that are incompatible with growth. These functions likely provide fitness effects that escape detection in assays of reproductive capacity routinely employed to assess evolutionary fitness, or to identify genes that confer stress-resistance in cycling cells.

## Background

Starvation of budding yeast cells by nutrient depletion leads to the transcriptional induction or repression of a large number of genes [1-3] in concert with a prolonged cell cycle arrest or exit from the cell cycle (reviewed in [4]; "starvation by nutrient depletion is subsequently referred to as "starvation" unless noted otherwise). In the absence of starvation conditions, many of the 2213 genes induced between 8 hours and 28 days of medium depletion according to Gasch et al. [1] are likely repressed by the Origin Recognition Complex (ORC), which is also required for initiation of DNA replication and for silencing genes in the silent mating type loci [5]. Other proteins likely participate in the chromatin-mediated repression of starvation-induced genes as well. For example, 49% the 2213 genes induced by starvation in the Gasch et al. experiments [1] are also induced by (in addition to the *orc2-1 *mutation in the second subunit of ORC [5]), deletion of genes encoding Tup1p [6] or the Sir2, Sir3 and Sir4 proteins [7], depletion of histone H4 [7], deletion of *SUM1 *[8], the *abf1-1 *mutation [9] or by a mutation in histone H3 [10], all of which disrupt gene silencing. Although some of the genes induced by these experimental manipulations may be upregulated indirectly downstream of the derepression of transcriptional activators, a substantial proportion are likely induced by disruption of chromatin-mediated transcriptional repression in the vicinity of these genes. Because many other proteins also participate in chromatin-mediated repression of genes, additional starvation-induced genes may be transcriptionally repressed in the absence of starvation conditions.

Genes encoding proteins with related functions are often clustered physically in eukaryotic genomes, for reasons that remain unclear [11-15]. In *S. cerevisiae*, genes induced by starvation or by the inactivation of various silencing proteins are non-randomly distributed in the genome, because they map near ORC binding sites in chromatin more frequently compared to all genes (20 – 32% vs. 16.4% of all genes) [5]. The 2020 genes repressed by starvation according to Gasch et al. [1] are also non-randomly distributed in the genome, but they map near ORC binding sites less frequently than most genes (12.1% vs. 16.4% of all genes) [5]. This suggests that many genes repressed by starvation were evolutionarily excluded from regions containing genes transcriptionally repressed by ORC. Monte Carlo-type simulations employing randomized genelists indicated that the non-random distribution of starvation-induced or -repressed genes in relation to ORC binding sites is highly unlikely to have occurred by chance [5]. Many of the genes induced by starvation are induced by other stresses as well [1,16]. We hypothesized that clustering of genes in the budding yeast genome is at least partly related to the non-random distribution of starvation and other stress-related genes in chromatin regions that are transcriptionally repressed by ORC and/or other proteins.

## Results

### Non-random clusters of starvation-related genes

**Figure 1:**
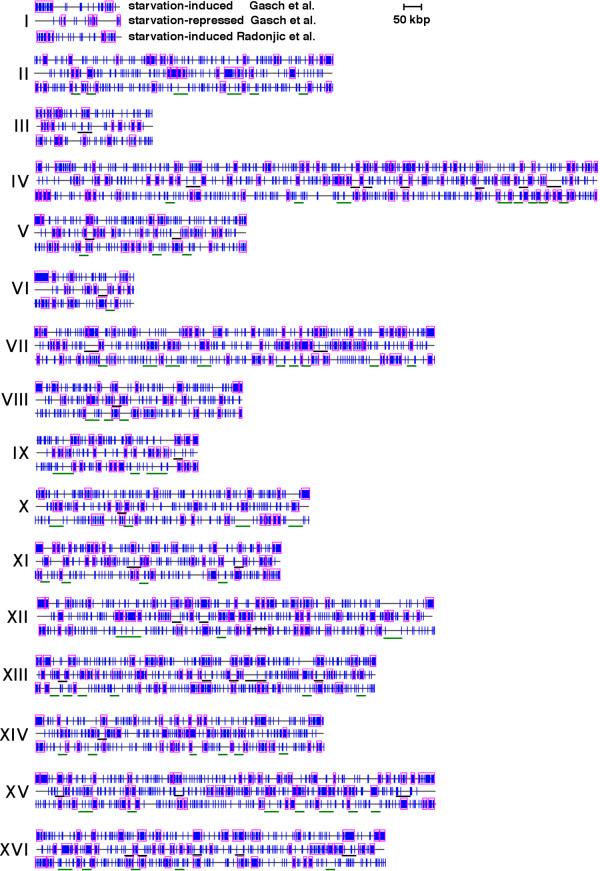
**Chromosome maps of *S. cerevisiae *genes induced or repressed during 8h-28d nutrient depletion according to Gasch et al. (2000) [1] or induced by 20 hours of nutrient depletion according to Radonjic et al (2005)**. Vertical blue lines indicate the position of genes induced or repressed in these datasets. Pink rectangles indicate clusters of genes detected by Pyxis that are unlikely to have formed by chance (p ≤ 0.05). Roman numerals indicate chromosome number. Relatively gene-free gaps in clusters of starvation-repressed genes that coincide with the map positions of many starvation-induced genes and/or gene clusters are underlined in black. Relatively gene-free gaps in maps of starvation-induced genes that contain many starvation-repressed genes and/or gene clusters are underlined in green

**Figure 2:**
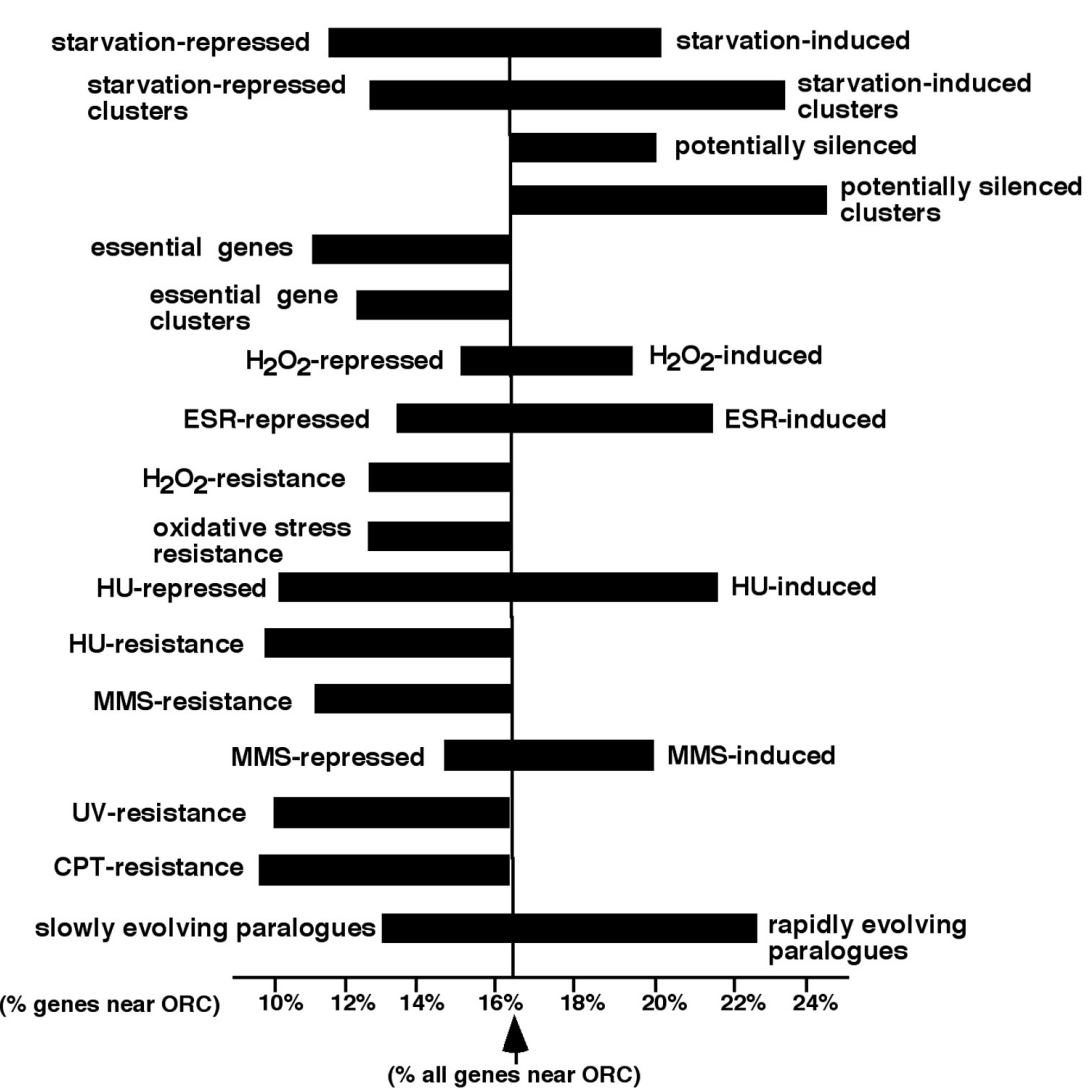
**Non-random distribution of stress-related genes in relation to ORC binding sites**. Ends of horizontal bars indicate the percent of genes in indicated datasets that map within 1 kb of potential ORC binding fragments detected in the budding yeast genome [18]. Vertical line demarks the 16.4% all genes that similarly map near an ORC binding site. Starvation-induced and starvation-repressed datasets are from Gasch et al. (2000) [1]. Other datasets are referenced in Materials and Methods.

To test this hypothesis, we systematically analyzed datasets of genes induced or repressed by starvation and other datasets using the Pyxis program, which detects physical clusters of genes that are unlikely to have formed by chance (p ≤ 0.05) [17]. Pyxis detected 189 non-random gene clusters in the dataset of 2213 genes induced 2-fold or more by starvation according to Gasch et al. [1]. (Fig. 1; "starvation-induced Gasch et al."). These clusters contain a total of 2.3 × 10^6 ^bp DNA and 1404 genes, 1068 of which are induced by starvation. Genes in these clusters map within 1 kb of previously detected ORC binding sites [18] even more frequently compared to all starvation-induced genes identified by Gasch et al. (Fig. 2; calculations of the statistical significance (based on Monte-Carlo simulations) of proximity to ORC binding sites for genes in this and related datasets are summarized in Additional File 1; comparisons between different datasets are summarized in Table 1). Clusters of starvation-induced genes also contain 205 (39%) of the 524 genes induced by the *orc2-1 *mutation, which likely disrupts the ORC-dependent repression of many of these genes [5]. The probability that the overlap between these datasets would occur by chance is very small (p < 10^-10^; statistical calculations for overlapping datasets are summarized in Additional File 2; in subsequent descriptions of overlaps between datasets, the probability that they would occur by chance is, in all cases, less than 10^-4 ^unless noted otherwise). Non-random clusters of starvation-induced genes are particularly prominent in telomere-proximal regions (Fig. 1; "starvation-induced Gasch et al."), which are enriched for ORC binding sites [18] as well as genes induced by stresses that reside within hypoacetylated chromatin [19]. In fact, the Pyxis program detected starvation-induced clusters of genes in 27 of the 32 telomere-proximal (within 50 kb of telomeres) regions of budding yeast chromosomes.

**Figure 3:**
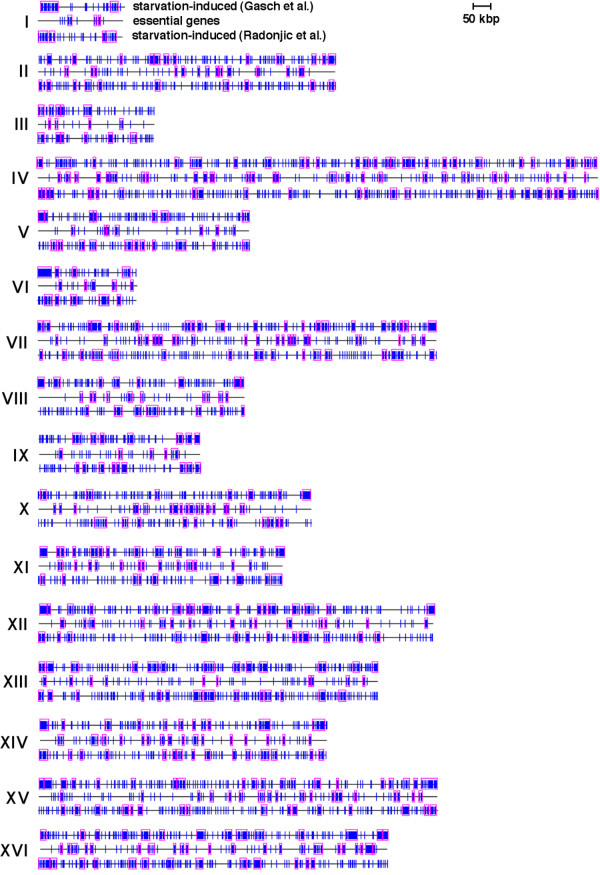
**Most genes essential for growth map between clusters of starvation-induced genes**. Vertical blue lines indicate the position of genes in starvation-induced or essential gene datasets. Pink rectangles indicate clusters of genes detected by Pyxis that are unlikely to have formed by chance (p ≤ 0.05). Roman numerals indicate chromosome number.

**Table 1:** Non-random distribution of stress-related genes in budding yeast

**Dataset**	**Number of genes in dataset**	**Number of genes in dataset within 1 kb ORC binding site(% genes in dataset)**	**Number of genes in starvation-induced clusters (% genes in dataset)**	**Number of genes in starvation-repressed clusters (% genes in dataset)**	**Number of potentially silenced genes (% genes in dataset)**
**all genes**	7104	1164 (16.4%)			
***orc2-1*-induced genes**	524	151 (28.8%) (+)	205 (38.9%) (+)	43 (8.0%) (-)	
**potentially silenced genes**	2111	431 (20.4%) (+)	644 (30.5%) (+)	238 (11.3%) (-)	2111 (100%)
					
***Starvation and other stress-related genes***					
**starvation-induced genes**	2213	443 (20.0%) (+)	1073 (48.5%) (+)	137 (6.2%) (-)	1086 (51.4%) (+)
**starvation-induced clusters**	1404	323 (23.1%) (+)	1404 (100%)	32 (2.3%) (-)	644 (45.9%) (+)
**starvation-repressed genes**	2020	224 (12.1%) (-)	105 (5.2%) (-)	1091 (54.0%) (+)	296 (19.6%) (-)
**starvation-repressed clusters**	1416	185 (13.0%) (-)	32 (2.3%) (-)	1416 (100%)	238 (16.8%) (-)
**starvation-induced genes†**	2042	400 (20%) (+)	676 (33.1%) (+)	148 (7.2%) (-)	999 (49.0%) (+)
**MMS)-induced genes**	452	93 (20.6%) (+)	181 (40.0%) (+)	55 (12.2%) (-)	210 (46.4%) (+)
**MMS)-repressed genes**	217	33 (15.2%*) (-)	20 (9.2%) (-)	87 (40.1%) (+)	26 (12.0%) (-)
**HU-induced genes**	116	24 (20.5%*) (+)	41 (35.3%) (+)	14 (12.1%) (-)	62 (53.4%) (+)
**HU-repressed genes**	71	7 (9.8) (-)	14 (19.7%*) (-)	29 (40.8%) (+)	21 (29.6%*) (-)
**H**_2_**0**_2_**-induced genes**	1379	255 (18.5%*) (+)	434 (31.5%) (+)	167 (12.1%) (-)	598 (43.4%) (+)
**H**_**2**_**0**_**2**_**-repressed genes**	1536	235 (15.3%) (-)	224 (14.6%) (-)	457 (29.8%) (+)	479 (31.1%*) (+)
**ESR-induced genes**	282	63 (22.3%) (+)	123 (43.6%) (+)	14 (5.0%) (-)	153 (54.2%) (+)
**ESR-repressed genes**	585	75 (12.8%) (-)	14 (2.4%) (-)	322 (55%) (+)	32 (5.5%) (-)
					
***Essential growth genes and genes required for stress resistance in cycling cells***					
**Genes essential for growth**	1106	130 (11.8%) (-)	106 (9.6%) (-)	355 (32.1%) (+)	161 (14.6%) (-)
**MMS-resistance**	103	11 (10.7%) (-)	6 (5.8%) (-)	20 (19.4%*) (-)	n.d.
**Camptothecin resistance**	83	8 (9.6%) (-)	7 (8.4%) (-)	14 (16.9%*) (-)	n.d.
**HU-resistance**	136	13 (9.6%) (-)	9 (6.6%) (-)	28 (20.6%*) (+)	n.d.
**H**_**2**_**0**_**2**_**-resistance**	123	15 (12.2%) (-)	13 (10.6%) (-)	24 (19.5%*) (-)	n.d.
**Oxidative stress resistance**	685	87 (12.8%) (-)	95 (13.9%) (-)	158 (23.0%*) (+)	n.d.
**UV-resistance**	307	34 (11.1%) (-)	30 (9.8%) (-)	56 (18.2%) (-)	n.d.
**All stress-resistance**	974	115 (11.8%) (-)	129 (13.2%) (-)	208 (21.3%*) (+)	251 (25.8%) (-)
					
***Slowly and rapidly evolving paralogues***					
**slowly evolving paralogues**	115	15 (13.0%) (-)	22 (19.1%*) (-)	34 (29.6%*) (+)	19 (16.5%) (-)
**rapidly evolving paralogues**	115	26 (22.6%) (+)	30 (26.0%*) (+)	16 (13.9%) (-)	57 (49.6%) (+)

(+) indicates more than expected by chance (-) indicates less than expected by chance n.d. = not determined

*denotes values that are not significantly different compared to values expected by chance. All other values are statistically significant (p ≤ 0.05).

†Starvation-induced dataset from Radonjic et al. [3] – other starvation datasets are from Gasch et al. (2000) [1]. Additional information for datasets is provided in Materials and Methods.

Pyxis detected a slightly larger number of non-random gene clusters (217) in the list of 2020 genes that are repressed, rather than induced, 2-fold or more by starvation according to Gasch et al. [1] (Fig. 1; "starvation-repressed Gasch et al."). These clusters contain a total of 2.4 × 10^6 ^bp DNA and 1416 genes, 1086 of which are repressed by starvation. Therefore, starvation-induced and starvation-repressed gene clusters identified in the datasets of Gasch et al. are similar in their overall lengths and number of genes. However, in contrast to the 23% of all genes that map within 1 kb of an ORC binding site in clusters induced by starvation, just 12% of the genes in starvation-repressed clusters similarly map near ORC binding sites (Fig. 2). Moreover, whereas 39% of the 524 genes induced by the *orc2-1 *mutation are found in starvation-induced clusters, starvation-repressed clusters contain just 8% of these genes. This is significantly less than expected by chance (p < 10^-10^). Just one of the clusters of starvation-repressed genes (on the right arm of chromosome I) resides in a telomere-proximal region. In fact, in contrast to the large number of starvation-induced genes in telomere-proximal regions of most chromosomes, genes repressed by starvation according to Gasch et al. are almost completely absent from these regions (Fig. 1; "starvation-repressed Gasch et al.").

**Figure 4:**
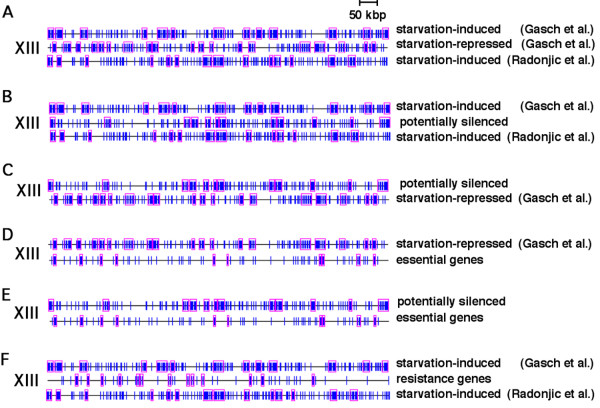
**Gene maps of chromosome XIII are representative of the relationship between clusters of genes in the various datasets indicated in each panel**. Vertical blue lines indicate the position of genes in the indicated datasets. Pink rectangles indicate clusters of genes detected by Pyxis that are unlikely to have formed by chance (p ≤ 0.05). Roman numerals indicate chromosome number. Additional Pyxis maps for all chromosomes are available in additional files 3, 4, 7.

A mutually exclusive relationship between clusters of genes induced or repressed by starvation was detected in internal regions of chromosomes as well – that is, non-random clusters of genes repressed by starvation in internal regions also frequently map to regions of chromosomes that contain very few genes induced by starvation, and vice versa (Fig. 1; compare "starvation-induced Gasch et al." with "starvation-repressed Gasch et al."). The reciprocal relationship between chromosome regions containing genes in these and other datasets are especially apparent in maps of chromosome XIII (Fig. 4). Overall, just 105 (5%) of the 2020 genes repressed by starvation map within one of the 189 clusters of genes induced by starvation in the Gasch et al. datasets, and only 137 (6%) of the 2213 genes induced by starvation map within one of the 217 clusters of genes repressed by starvation. Furthermore, despite the fact that genes in both sets of clusters (starvation-induced or repressed) include approximately half of all budding yeast genes, clusters of starvation-repressed genes rarely overlap clusters of starvation-induced genes – just 32 genes are found in both sets of clusters.

The mutually exclusive relationship between starvation-induced or -repressed gene clusters was also apparent in a comparison of the Pyxis map of starvation-repressed genes detected by Gasch et al. with maps of a second, independently derived dataset of genes induced by starvation recently reported by Radonjic et al. [3]. This latter dataset contains 2042 genes that were induced and 4035 genes that were repressed at 20 hours of medium depletion compared to mid-log phase cultures. Extensive overlap exists between the Radonjic et al. and Gasch et al. datasets of starvation-induced genes, including within clusters of these genes. For example, 33% of the genes induced by starvation according to Radonjic et al. are found in clusters of starvation-induced genes in the Gasch et al. dataset. In contrast, just 7.2% of starvation-induced genes identified by Radonjic et al. are in clusters of genes found to be repressed by starvation by Gasch et al. Similar to the Gasch et al. dataset of genes induced by medium depletion starvation, genes induced by starvation in the Radonjic et al. study map near ORC binding sites more frequently than all genes (Table 1).

814 of the genes induced by starvation in the Radonjic et al. experiments were detected by Pyxis in non-random clusters that frequently coincided with, or overlapped, clusters of genes induced in the Gasch et al. experiments (Fig. 1; compare "starvation-induced Gasch et al." with "starvation-induced Radonjic et al."). In contrast, clusters of starvation-induced genes detected by Pyxis in the Radonjic et al. dataset rarely overlap clusters of genes repressed by starvation in the Gasch et al. dataset – just 21 genes are found in both sets of these clusters. In both telomere-proximal and internal regions of chromosomes, relatively gene-free gaps in chromosomal maps of starvation-repressed genes frequently coincide with regions containing many genes and/or clusters of genes from both independently derived datasets of starvation-induced genes (in Fig. 1, many of these gaps in internal regions of chromosomes are underlined in black). Conversely, relatively gene-free gaps in chromosomal maps of both starvation-induced datasets often coincide with regions containing many starvation-repressed genes and/or clusters of these genes (many of these latter gaps are underlined in green in Fig. 1). Therefore, the mutually exclusive relationship between clusters of starvation-induced and -repressed genes detected throughout budding yeast chromosomes is not specific to a single dataset of starvation-regulated genes.

### Many starvation-induced genes are likely silenced in the absence of stress

In some cases, one boundary of starvation-induced clusters in both datasets of starvation-induced genes maps precisely (within a few genes) to the boundary of an adjacent cluster of genes repressed by starvation. Occasionally, both the left and right boundaries of clusters of starvation-induced genes coincide with the boundaries of adjacent clusters of starvation-repressed genes on either side, or vice versa (Figs. 1, 4A). The shared boundaries between clusters of starvation-induced and starvation-repressed genes suggest that some of these clusters correspond to differentially regulated chromatin domains. Consistent with this possibility, 46% of the 1404 genes that reside in starvation-induced clusters detected in the Gasch et al. datasets are also found in the list of 2111 genes induced by mutations or other experimental manipulations described above that inactivate proteins required for establishing a repressive chromatin environment (these genes are subsequently referred to as "potentially silenced"). Similarly, 51% of all 814 genes in clusters detected in the Radonjic et al. dataset are potentially silenced as defined in this fashion. Both percentages are significantly higher than the percentage of all genes that are potentially silenced (which is approximately 30%). In contrast, just 17% of the 1416 genes that reside in starvation-repressed clusters are potentially silenced. The size of the overlaps between genes in starvation-induced and -repressed clusters identified in the Gasch et al. datasets and the list of potentially silenced genes is significantly larger (in the case of starvation-induced genes) or smaller (in the case of starvation-repressed genes) than expected by chance (p < 10^-10^).

Non-random clusters of genes were also detected by Pyxis in the list of 2111 potentially silenced genes, and these clusters often overlap Pyxis-detected clusters of starvation-induced genes, especially in telomere-proximal regions (Figs. 4B and Additional File 3; compare "potentially silenced" with "starvation-induced Gasch et al." maps). In contrast to the small overlap between genes in starvation-repressed clusters and either dataset of starvation-induced clusters (32 genes in the Gasch et al. and 21 in the Radonjic et al. datasets), 842 (74%) of the 1139 genes in clusters of potentially silenced genes also reside in starvation-induced clusters from either the Gasch et al. or Radonjic et al. datasets. Clusters of potentially silenced genes overlap clusters of starvation-repressed genes far less frequently and exhibit a mutually exclusive relationship similar to that between starvation-induced and -repressed clusters (Figs. 4C and Additional File 4; compare "potentially silenced" with "starvation repressed"). In fact, clusters of genes repressed by starvation contain just 81 of the 1139 genes detected in clusters of potentially silenced genes. This is less than a third of the number of genes expected in this overlap by chance (p < 10^-10^). Therefore, clusters of starvation-induced genes are enriched for genes that are potentially silenced, and potentially silenced genes are underrepresented in gene clusters that are repressed by starvation.

### Clusters of genes induced or repressed by starvation reside within chromatin regions differentially regulated by the histone deacetylase Hda1p

Chromatin repression of genes is mediated in part by reduced acetylation of histones. The histone deacetylase Hda1p likely represses transcription of a large number of *S. cerevisiae *genes by deacetylating the histones H2B and H3 in the vicinity of these genes. These genes include many starvation and other stress-induced genes residing in telomere-proximal regions [19], which also frequently harbor non-random clusters of starvation-induced genes detected by Pyxis. To determine whether differences in the potential for silencing of genes in starvation-induced compared to starvation-repressed clusters in these and other regions of chromosomes might be related to the differential regulation of histone acetylation by Hda1p in the vicinity of these genes, we compared the distribution of these starvation-related genes with the distribution of genes previously reported to be enriched for histone H3 acetylated on K18 (which is deacetylated by Hda1p) in a strain deleted of *HDA1 *compared to a congenic wild type strain [19].

**Figure 5:**
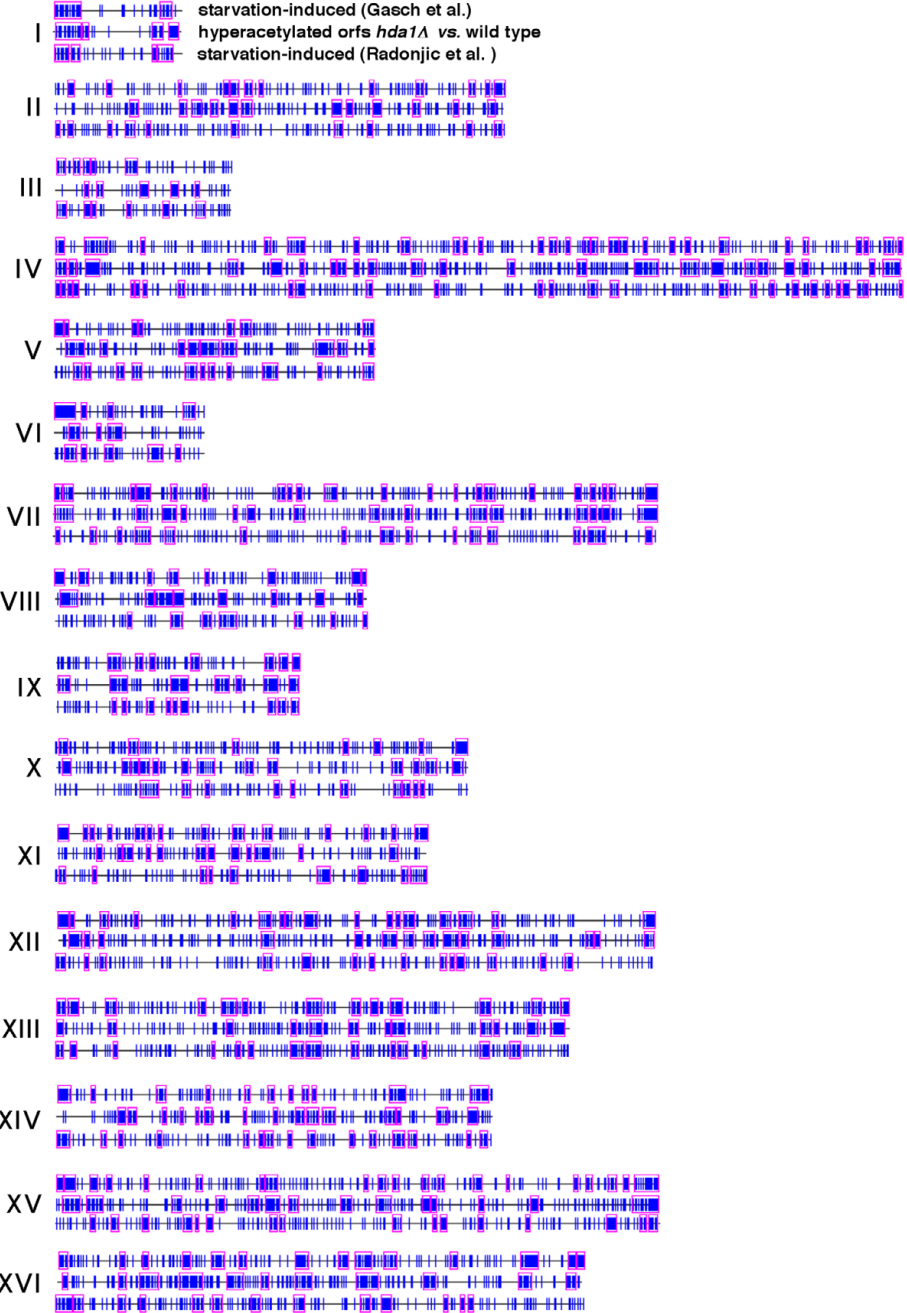
**Clusters of genes induced by starvation frequently overlap clusters of genes that are likely regulated by the histone deacetylase Hda1p**. Vertical blue lines indicate the position of genes induced by starvation (top and bottom gene maps for each chromosome) or that are enriched for histone H3 acetylated on K18 in *hda1*Δ compared to wild type cells (middle gene map for each chromosome). Pink rectangles indicate clusters of genes detected by Pyxis in each dataset that are unlikely to have formed by chance (p ≤ 0.05). Roman numerals indicate chromosome number.

**Figure 6:**
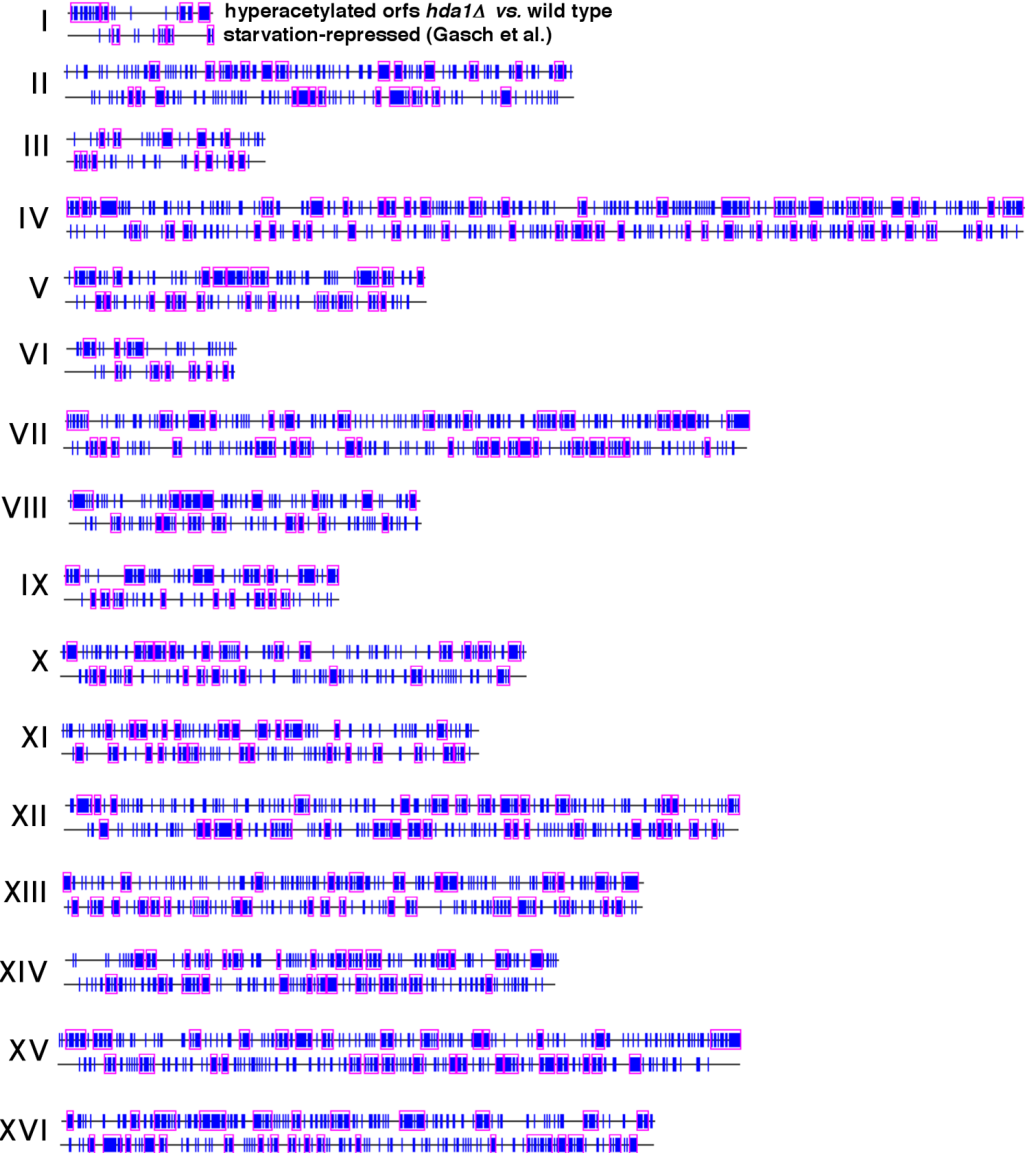
**Clusters of genes repressed by starvation less frequently contain genes enriched for histones deacetylated by Hda1p**. Vertical blue lines indicate the position of genes repressed by starvation (bottom gene map for each chromosome) or that are enriched for histone H3 acetylated on K18 in *hda1*Δ compared to wild type cells (top map for each chromosome). Pink rectangles indicate clusters of genes detected by Pyxis in each dataset that are unlikely to have formed by chance (p ≤ 0.05). Roman numerals indicate chromosome number.

Many of the 2418 genes acetylated in *hda1*Δ compared to wild type cells [19] reside in non-random clusters detected by Pyxis that frequently overlap clusters detected in both the Gasch et al. and Radonjic et al. datasets of starvation-induced genes. This overlap is particularly apparent in telomere-proximal regions (Fig. 5). Overall, 110 of the 189 starvation-induced clusters detected in the Gasch et al. datasets partially or completely overlap clusters of genes that are deacetylated by Hda1p. In some cases, the boundaries of clusters detected in all three datasets coincide (Fig. 5). In contrast, clusters of genes hyperacetylated in *hda1*Δ compared to wild type cells much less frequently overlap clusters of starvation-repressed genes (Fig. 6). Just 40 of the 217 clusters of starvation-repressed genes in the Gasch et al. dataset overlap clusters of genes that are hyperacetylated in *hda1*Δ compared to wild type cells.

The fraction of genes in the 189 starvation-induced clusters detected in the Gasch et al. datasets that are hyperacetylated in *hda1*Δ compared to wild type cells is 57%. This is significantly higher than the ~40% of all genes that are hyperacetylated in *hda1*Δ compared to wild type cells (p < 10^-11^; statistical calculations for overlaps between various datasets and the dataset of genes hyperacetylated in *hda1*Δ compared to wild type cells are summarized in Additional File 5). Although telomere-proximal regions are enriched for starvation- and other stress-induced genes maintained in a hypoacetylated state by Hda1p [19], this cannot account for the overall increase in acetylation of genes within starvation-induced clusters in *hda1*Δ compared to wild type cells. This is because the frequency with which genes in internal (>50 kb from telomeres) starvation-induced clusters are acetylated in *hda1*Δ compared to wild type cells is similar to telomere-proximal clusters of genes (57% of genes in internal clusters vs. 59% in telomere-proximal clusters).

**Figure 7:**
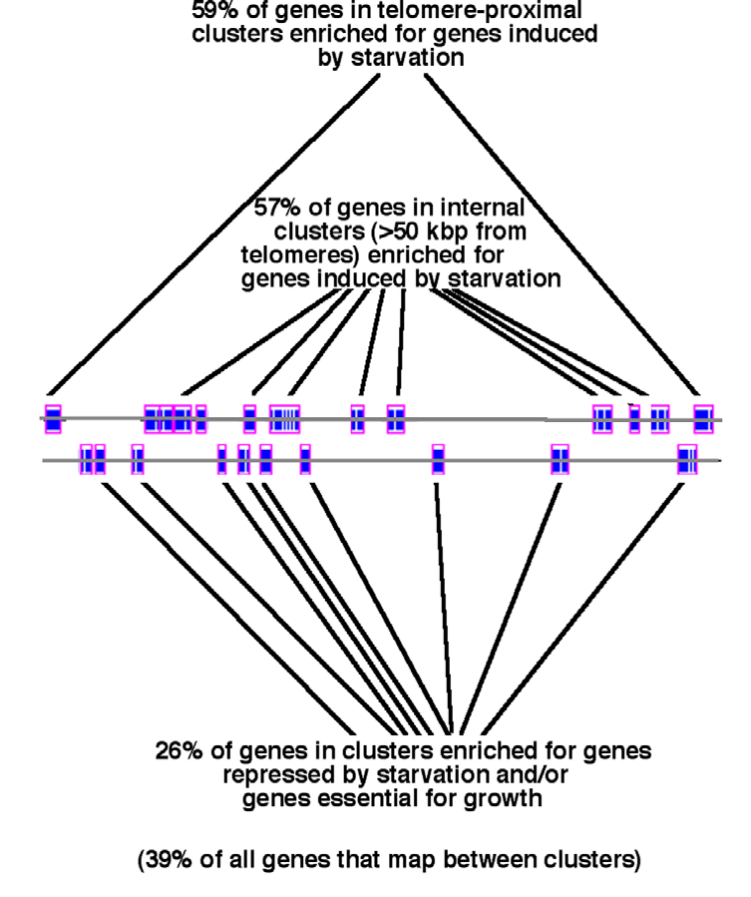
**Summary of the frequency with which genes in various datasets are enriched for histones deacetylated by Hda1p in cycling cells**. Percentages indicate the fraction of genes in each dataset that are enriched for histone H3 acetylated on K18 in *hda1*Δ compared to wild type cells (which are therefore deacetylated by Hda1p in wild type cells). "Genes not in clusters" refers to the dataset of all genes that are not found in starvation-induced or -repressed clusters according to Gasch et al. (2000) or in clusters of genes that are essential for growth.

In contrast, genes in the 217 starvation-repressed clusters detected in the Gasch et al. datasets are much less frequently acetylated in *hda1*Δ compared to wild type cells (25.5%). The frequencies with which genes in either starvation-induced (57%) or -repressed (25.5%) clusters are deacetylated by Hda1p is significantly different from the frequency with which all other genes residing outside of starvation-induced or -repressed clusters are deacetylated, which is 39%. Therefore, starvation-induced clusters are enriched for genes that are likely repressed in cycling cells by the action of Hda1p. Conversely, genes in starvation-repressed clusters are less likely (compared to all genes) to be maintained silent by this protein in cycling cells (these and other findings related to histone acetylation described below are summarized in Fig. 7 and Additional File 5). This analysis employed histone acetylation data from Robyr et al. [19] specific for histones associated with DNA sequences in ORFs rather than intergenic regions containing gene promoters, where deacetylation of histones by Hda1p occurs to a greater extent. We reasoned that deacetylation by Hda1p of histones within ORF sequences, rather than promoter regions, might better reflect the existence of extended chromatin domains. However, a similar pattern of changes in the acetylation state of intergenic sequences occurred in starvation-induced and -repressed clusters compared to all genes (Additional File 6).

### Non-random distribution of genes induced or repressed by other stresses

Genes induced or repressed by other stresses are also distributed non-randomly in the budding yeast genome. For example, of the 452 genes induced 2-fold or more by exposure to the DNA-damaging agent methyl methanesulfonate (MMS) according to Gasch et al. (2001) [20], 181 (40%) reside in starvation-induced clusters detected in the Gasch et al. (2000) datasets, and only 55 (12%) reside in starvation-repressed clusters. Conversely, 87 (40%) of the 217 genes repressed 2-fold or more by MMS map within clusters of genes down-regulated by starvation, and only 20 (10%) of these 217 genes map within clusters induced by starvation. This is consistent with reports of extensive overlap between datasets of genes induced or repressed by starvation or MMS [16]. Furthermore, similar to starvation-induced or -repressed genes, genes induced or repressed by MMS are non-randomly distributed in the genome in relation to ORC binding sites. Although 21% of the 452 genes induced 2-fold or more by MMS map within 1 kb of an ORC binding site, only 15% of the 217 genes repressed by MMS map near an ORC binding site (Fig. 2). As with the starvation-induced dataset, based on Monte Carlo simulations, the increased number of MMS-induced genes that map near ORC binding sites compared to all genes is statistically significant (p < 10^-2^).

A similar reciprocal relationship exists between genes induced or repressed by the replication stress-inducing drug hydroxyurea (HU) [21], the oxidative stress-inducing compound hydrogen peroxide (H_2_0_2_) [1], or as part of the generic Environmental Stress Response (ESR) [1]. In each case, compared to all genes, genes induced by these conditions more frequently map near ORC binding sites, reside in clusters of starvation-induced genes and are potentially silenced, and less frequently reside in clusters of genes repressed by starvation. In contrast, genes repressed by each of these conditions less frequently map near ORC binding sites compared to all genes, are more frequently found in clusters of starvation-repressed genes, and less frequently reside in clusters of starvation-induced genes or in the list of potentially silenced genes (Fig. 2; Table 1).

### Non-random distribution of genes essential for growth

The findings described above are consistent with the hypothesis that an important factor in the evolution of gene clustering in the *S. cerevisiae *genome has been chromatin-mediated repression (in the absence of stress) of genes that are induced by starvation and other stresses. The exclusion of starvation-repressed genes from regions of repressive chromatin may reflect a requirement for the expression of these genes in cycling cells. Genes expressed in cycling cells include ~1106 budding yeast genes essential for growth in rich medium [22] ("essential genes"), which were previously shown to cluster non-randomly in the budding yeast genome [23]. Pyxis also detected a large number of essential genes (722) in non-random clusters containing a total of 1467 genes. Similar to starvation-repressed gene clusters, clusters of essential genes are almost completely absent from telomere-proximal regions (Fig. 3). Furthermore, 620 (56%) of all essential genes are repressed by starvation according to Gasch et al., which is 255 genes more than expected randomly (p < 10^-10^). In contrast, just 136 (12%) of all 1106 essential genes were induced by starvation in the Gasch et al. experiments, and only 106 (9.6%) of all essential genes were found in Pyxis-detected clusters of starvation-induced genes (p < 10^-10^). Similar to starvation-repressed genes, essential genes and essential gene clusters often map between clusters of genes induced by starvation or in other regions of chromosomes that harbor very few starvation-induced or potentially silenced genes (Figs. 3 and 4E). Essential genes and essential gene clusters more frequently overlap clusters of starvation-repressed genes (Fig. 4D).

Also similar to genes repressed by starvation, essential genes less frequently are induced by inactivation of the silencing proteins described above (17% vs. 49% of all starvation-induced genes in the Gasch et al. datasets). Histone H3 molecules associated with essential genes are also less frequently deacetylated by Hda1p, and at a frequency comparable to genes in starvation-repressed clusters (25% vs. 40%; Additional File 5). Essential genes also map near ORC binding sites less frequently than most genes (Fig. 2), and this difference is highly significant (p = 10^-4^). Therefore, similar to genes repressed by starvation, clustering of genes essential for growth likely reflects at least in part their exclusion from regions of repressive chromatin due to a requirement for their expression in cycling cells.

### Non-random distribution of genes that confer stress resistance in cycling cells

Genes that confer resistance to various stresses have been identified by reduced growth and/or viability in response to these stresses in cells deleted of these genes. Somewhat surprisingly, stress resistance genes identified by this approach infrequently coincide with genes transcriptionally induced by the same stresses. This may reflect a requirement for the constitutive expression of stress-resistance genes defined in this manner [16,24,25]. Similar to genes repressed by stress and/or essential for growth, a requirement for the expression of these genes may have lead to their evolutionary exclusion from regions of repressive chromatin. In fact, in contrast to genes that are transcriptionally induced by the same stress-inducing agents, deletion of genes that results in increased sensitivity to the DNA damaging agents MMS [24], UV radiation or the topoisomerase I inhibitor camptothecin [26], replication stress-inducing HU [26], or oxidative stress-inducing H_2_O_2 _and other oxidative stress-inducing agents [25] are all under-represented in clusters of starvation-induced genes (Table 1). Although none of these genes are essential for growth, similar to essential genes, an aggregate list of 974 genes that confer resistance defined in this fashion to one or more of these stresses are rarely found in telomere-proximal regions (Fig. 4F and Additional File 7) and are under-represented in the list of all potentially silenced genes (Table 1). Furthermore, although genes induced by the various stresses described above map near ORC binding sites more frequently than expected, in every case, genes that confer resistance to the same stresses map near ORC binding sites less frequently than expected (Fig. 2; in some cases, however, this under-representation is not statistically significant (Additional File 2)). Similar to genes essential for growth and genes repressed by starvation, genes that confer resistance to stresses often map between clusters of starvation-induced genes (Fig. 4F and Additional File 7). Histone H3 associated with stress-resistance genes is also less frequently deacetylated by Hda1p compared to genes residing in starvation-induced clusters (34% vs. 57%). This is consistent with the possibility that many of these genes also have been evolutionarily excluded from regions of repressive chromatin.

### Differential regulation of slowly versus rapidly evolving duplicated genes

*S. cerevisiae *underwent an ancient whole-genome duplication after diverging from the related yeast, *K. waltii *[27,28]. In 115 gene pairs that originated with this duplication, one of the two paralogues evolved significantly faster than the other after the duplication event [27]. The 115 more slowly evolving paralogues are more likely to have retained ancestral functions acquired before the whole-genome duplication and include 18 genes essential for growth. In contrast, many of the 115 more rapidly evolving paralogues of these gene pairs acquired derivative, more specialized functions, none of which is essential for growth [27]. These functional distinctions are similar to those associated with genes induced or repressed by starvation and other stresses, which are more or less likely to be silenced by chromatin in the absence of stress. In fact, one of the more rapidly evolving paralogues encodes the histone deacetylase Sir3p, which is derived from the DNA-binding function of Orc1p encoded by its more slowly evolving paralogue [27]. This suggests that the less essential, more specialized functions of the rapidly evolving paralogues arose in parallel with more robust silencing by Sir3p and other proteins.

Consistent with this possibility, 57 of the 115 rapidly evolving paralogues are found in the list of 2111 genes that are potentially silenced. This is 19 more genes than expected on a random basis, and this over-representation is statistically significant (p < 3.8 × 10^-4^). In contrast, just 19 of the 115 more slowly evolving paralogues that arose from the whole genome duplication are potentially silenced. This is half the number expected by chance, and this under-representation is statistically significant (p < 6.8 × 10^-6^). None of the 57 potentially silenced, rapidly evolving paralogues is a duplicate of a slowly evolving paralogue that is also potentially silenced. Therefore, the potential for silencing of all 57 of these genes likely arose after the whole-genome duplication in parallel with the evolution of new or derived functions. A role for ORC in silencing of some of these genes is suggested by the fact that the 57 rapidly evolving paralogues that are also potentially silenced include all 21 of the 115 more rapidly evolving paralogues that are also induced by the *orc2-1 *mutation [5]. In contrast, just 3 of the 115 slowly evolving paralogues are induced by the *orc2-1 *mutation. Furthermore, 26 (23%) of the 115 more rapidly evolving paralogues map near ORC binding sites (Fig. 2 and Table 1). Based on Monte Carlo-type simulations, this represents a statistically significant increase compared to the 15 (13%) of the 115 slowly evolving paralogues or the 16% of all genes that also map near ORC binding sites. Moreover, just 4 of the 26 more rapidly evolving paralogues that map near ORC are duplicates of a slowly evolving paralogue that also maps near an ORC binding site. Therefore, close proximity to ORC of at least 22 of these 26 paralogues either arose after the whole-genome duplication event, or was lost from their slowly evolving counterparts since this event.

An increased propensity for transcriptional repression of rapidly evolving paralogues is also suggested by the fact that 58 of these 115 paralogues are enriched for histones deacetylated by Hda1p, which is 17 more than expected by chance and this over-representation is also statistically significant (p < 0.001). In contrast, just 31 slowly evolving paralogues are enriched for histones deacetylated by Hda1p, which is 13 less than expected by chance, and this under-representation is statistically significant (p < 0.01). These findings suggest that the more derived, less essential functions of rapidly evolving paralogues arose after the whole genome duplication in parallel with an increased potential for silencing by ORC, Hda1p and other proteins. These functions include transcriptional responses to starvation, because 61 of the 115 rapidly evolving paralogues were also induced by starvation conditions in the Gasch et al. experiments. This is almost twice the number (34) of rapidly evolving paralogues that were repressed, rather than induced, by starvation in these experiments despite the similar number of genes induced or repressed by starvation overall. It is also 20 genes more than expected randomly, and this difference is significant. Furthermore, the 61 rapidly evolving paralogues induced by starvation include 42 of the 57 that are also potentially silenced, and 36 of the 58 rapidly evolving paralogues deacetylated by Hda1p. In addition, just 9 of the 115 more rapidly evolving paralogues are in the list of 974 genes that confer resistance to stresses. This is half the number expected by chance, and this difference is also statistically significant. It is also less than half of the 20 genes that confer resistance to stress found in the list of 115 slowly evolving paralogues. Therefore, the increased frequency with which rapidly evolving paralogues are likely silenced by ORC, Hda1p and other proteins may have evolved since the whole genome duplication in parallel with an increase in the number of these paralogues that are induced by starvation, but not in the number repressed by starvation or that are essential for growth or resistance to stresses.

## Discussion

Our findings suggest that a fundamental organizing principle during evolution of the *S. cerevisiae *genome has been clustering of genes induced by starvation and other stresses in chromatin regions that, in the absence of stress, are transcriptionally repressed, from which most genes expressed in cycling cells have been excluded. They are also consistent with the possibility that repression by ORC of at least some starvation and other stress-induced genes contributed to the evolutionary organization of these clusters. A role for chromatin structure in the evolution of gene clusters is not inconsistent with epistatic selection for genetic linkage in the evolution of gene clusters, as was recently proposed for the *DAL *gene cluster on chromosome IX [29]. Five of the six adjacent genes in this cluster – which evolved after *S. cerevisiae *diverged from *K. waltii *[29] – are coordinately induced between 8 hours and 28 days of medium depletion according to Gasch et al. [1] and four of the genes in this cluster are induced by 20 hours of medium depletion according to Radonjic et al. [3]. In addition to their regulation by catabolite repression [30], several genes in the *DAL *gene cluster are also likely repressed by the Hst1 deacetylase and the transcriptional repressor Sum1p ([29] and references therein), both of which have been implicated in ORC-dependent repression of genes. In fact, several genes in the *DAL *gene cluster are induced by the *orc2-1 *mutation, and at least two of these genes are also induced by  deletion of nearby ORC binding sites, and not by the *orc2-1* mutation when these binding sites are deleted from *orc2-1* cells [5]. Therefore, at least some of the genes in the *DAL *gene cluster are likely repressed by ORC. As was proposed by Wong and Wolfe [29], a repressive chromatin environment in the *DAL *gene cluster presumably conferred a selective advantage that contributed to the maintenance of these genes in linkage disequilibrium.

Genes that appear to have been evolutionarily excluded from starvation-induced gene clusters identified by Pyxis in both the Gasch et al. and Radonjic et al. datasets include most (~86%) of the genes previously identified as essential for growth in rich medium, as well as a large fraction (~80%) of the 974 genes in our composite dataset of genes required for maintaining growth or viability of cycling cells exposed to various stresses. This is substantially more genes (approximately 2-fold more) than expected on a random basis. Genes that are essential for growth must, of course, be expressed in cycling cells, and exclusion of many of these genes from regions where genes may be repressed by chromatin structure is not unexpected. The exclusion from these same regions of many stress-resistance genes identified by increased sensitivity to stresses of strains deleted of these genes points to a similar requirement for the expression of these genes in cycling cells. As was proposed earlier [16,24,25], the constitutive expression of these genes may facilitate rapid responses to stress via post-transcriptional regulatory mechanisms. In the context of the more stressful natural environment in which *S. cerevisiae *evolved compared to rich medium in laboratory flasks, the proteins encoded by these genes may be equally indispensable for maintaining viability in natural populations of cycling cells.

At least over shorter evolutionary distances, a significant correlation exists between protein dispensability and evolutionary rate [31,32]. This correlation is reflected in the absence of genes that are essential for growth from the list of 115 rapidly evolving paralogues that arose during the whole genome duplication, compared to the 18 slowly evolving counterparts of these genes that are essential for growth [27]. The increased frequency with which rapidly, compared to slowly evolving paralogues that arose during the whole genome duplication are also induced by starvation and/or repressed by ORC and other proteins and the fact that most (~86%) genes induced by starvation are dispensable suggests that, in general, starvation and other stress-induced genes evolved more rapidly in parallel with less essential functions in cycling cells and the evolution of mechanisms for repressing these genes in the absence of stress. Two important questions, then, are: 1) why are many genes induced by starvation and other stresses repressed when stress is absent; and 2) when induced by stress, how do the less essential, more specialized functions of these genes contribute to fitness in the face of a stressful environment?

In the case of starvation-induced genes, the answer to the first question is reasonably straightforward. The induction of genes by starvation is accompanied by a prolonged G1 arrest and/or entry into a quiescent state [4]. In fact, in the natural environment in which they evolved, budding yeast more frequently exist in a quiescent state, rather than a state of growth [33]. Presumably, relaxed selective constraints associated with the whole genome duplication and other factors allowed for the evolution of genes encoding proteins required for prolonged cell cycle arrest and/or quiescence instead of growth. However, the evolution of genes that, when expressed, produce proteins whose functions are incompatible with growth would require the parallel evolution of mechanisms for ensuring that these genes are not expressed in unstressed cycling cells. Interestingly, in the absence of starvation conditions, a significant inverse relationship exists between protein dispensability and expression levels in cycling populations of budding yeast cells, and this inverse relationship is independent of the relationship between dispensability and evolution rate [31,32]. The basis for this inverse relationship may be transcriptional repression of a large number of rapidly evolving starvation-induced genes that are not only dispensable for growth, but cannot be expressed in cycling cells.

Less clear is how genes induced by starvation and other stresses confer evolutionary fitness. There are numerous reports that, unlike stress-resistance genes defined by increased sensitivity to stresses of cycling cells deleted of these genes, deletion of budding yeast genes transcriptionally induced by a variety of stresses does not alter the sensitivity of cycling populations of these cells to these stresses [16,24,25,34,35]. For example, it has been proposed that transcriptional responses play little, if any, role in modulating sensitivity to MMS [24] and other DNA-damaging agents [16]. This is not consistent, however, with resistance to a variety of stresses – including DNA damage induced by MMS and other DNA damaging agents – reported in starved or otherwise stressed cells in association with the induction of a large number of stress-related genes (reviewed in [4]). Nor is it consistent with the significant overlap between lists of genes and regulatory pathways induced by starvation or other stresses, including DNA damage [1,2,20,35]. The evolutionary organization of genes induced by a variety of stresses in non-random gene clusters strongly points to increased fitness associated with a general transcriptional response to stress coordinated at the level of chromatin structure.

These fitness effects likely include a G1 arrest that blocks entry into S phase under starvation and other stress conditions that may be sub-optimal for replicating chromosomes. Consistent with this model, cells harboring the *mec1-21 *mutation that causes defects in cellular responses to DNA replication stress [36] rapidly lose viability when starved by nutrient depletion in concert with an inefficient exit from S phase, but not when *RNR1*, the product of which is required for dNTP synthesis, and thus DNA replication, is overexpressed (M. Weinberger and W. Burhans; unpublished). Therefore, starved cells are subject to replication stress. Although it remains unclear whether other stresses impact on the DNA replication machinery, in addition to starvation [4], stresses including heat [37], oxidative stress [38], DNA damage [39] and high osmolarity [40] all induce at least a transient G1 arrest. Furthermore, abrogation of osmotic stress-induced G1 arrest leads to genetic instability in association with entry into S phase [40], which strongly suggests that osmotic stress can also induce replication stress.

G1 arrest induced by all these stresses likely requires downregulation of the *CLNI *and *CLN2 *genes encoding proteins required for progression through Start in G1. Transcription of *CLN1 *and *CLN2 *are repressed by multiple mechanisms in response to stresses. These include the Hog1 pathway that responds to osmotic stress [40] and repression by the product of the *XBP1 *gene, which is transcriptionally induced by a variety of stresses, including osmotic stress and starvation [41]. *XBP1 *transcripts are barely detectable in cycling cells [41]. Interestingly, *XBP1 *resides within a non-random starvation-induced cluster of genes, overlaps an ORC binding site and is induced by the *orc2-1 *mutation [5]. This suggests that, in the absence of stress, ORC participates in the repression of *XBP1 *and perhaps other genes in this cluster. When induced by stresses, the subsequent G1 arrest mediated by Hog1p, Xbp1p and other proteins could contribute to the cross-resistance to a variety of other stresses frequently observed in starved or otherwise stressed cells. The reduced expression in cycling cells of *XBP1 *and other stress-induced genes, redundancy in mechanisms of G1 arrest induced in response to stress by the proteins these genes encode, and the absence of a role for these genes in cell division provide a compelling explanation for why genes that are transcriptionally induced by stresses often escape detection in assays designed to detect effects of stresses on the growth of cycling populations of cells deleted of various genes.

In general, fitness effects of genes that are required for a quiescent state, rather than cell division, and are transcriptionally repressed in cycling cells would escape detection in assays that measure fitness by reduced growth of cells deleted of various genes. Consequently, they represent an important, but often ignored, factor determining evolutionary fitness in budding yeast. In the context of the lengthy time budding yeast cells likely spend in a stress-induced quiescent state in their natural environment, an equally important measure of fitness may be survival under starvation conditions.

In fact, increased fitness associated with the evolution of genes that promote quiescence, rather than reproduction, may be one of the more important consequences of whole-genome duplication events that occurred in many eukaryotes, including metazoans [42]. For example, the evolution of quiescence-promoting genes presumably was required for the evolution of differentiated states in metazoan tissues, where cells no longer divide. Mammalian cells are also driven out of the cell cycle by starvation and other stresses via a mechanism related to senescence, where they remain less sensitive to subsequent stresses [43]. Interestingly, in human cells, genes induced during senescence or repressed when entering quiescence are also often physically clustered [44]. Our findings provide a framework for understanding how quiescence is globally regulated by chromatin structure and other factors in budding yeast. This framework may illuminate some of the details of mechanisms underlying differentiation and stress resistance in mammalian cells, and how dysregulation of these mechanisms contribute to aging and diseases such as cancer.

## Methods

### Microarray datasets

Microarray datasets of starvation-related genes employed in this study include genes induced or repressed 2-fold or more during 8 hours to 28 days of medium depletion according to Gasch et al. (2000) [1] and genes induced or repressed 20 hours after refeeding of stationary phase cultures according to Radonjic et al. (2005) [3]. Expression values from this latter study corresponded to log ratios obtained by normalization to an external control. Genes induced or repressed by medium depletion starvation in the Radonjic et al. study were identified by subtracting log ratios at the 6.5 hour timepoint (mid-log phase growth) from log ratios at the 20 hour time point (which coincided with cessation of growth). The "potentially silenced" dataset corresponds to genes induced 2-fold or more after a 6 hour depletion of histone H4 or by deletion of the *SIR2*, *SIR3*, or *SIR4 *genes [7], deletion of the *TUP1 *gene [6], deletion of *SUM1 *[8], the *abf1-1 *mutation [9], a mutation in histone H3 [10] or by the *orc2-1 *mutation [5]. The "essential" growth gene dataset corresponds to genes required for growth in rich medium [22]. "HU-induced" and "HU-repressed" datasets correspond to genes induced or repressed by 1 hour treatment of cells with HU (hydroxyurea) [21]. "MMS-induced" and "MMS-repressed" datasets correspond to all genes induced or repressed by exposure to 0.02% MMS for 5' to 2 hours [20]. "HU-resistance", "MMS-resistance" "camptothecin-resistance" and "UV-resistance" datasets correspond to genes, the deletion of which confers sensitivity to hydroxyurea, MMS, camptothecin or UV radiation respectively [26]. "H_2_O_2_-resistance" dataset corresponds to genes, the deletion of which confers sensitivity to H_2_O_2_[25]. "H_2_O_2_-induced" and "H_2_O_2_-repressed" datasets correspond to genes induced or repressed between 5'-120' of H_2_O_2 _treatment [1]. "Oxidative stress-resistance" dataset corresponds to genes, the deletion of which confers sensitivity to a variety of different oxidative-stress-inducing agents [25]. "Slowly evolving paralogues" and "Rapidly evolving paralogues" correspond to gene pairs produced by an ancient whole-genome duplication event that are evolving at significantly different rates [27]. Changes in the acetylation state of K18 in histone H3 throughout the genomes of *hda1*Δ vs. wild type cells detected by ChIP microarrays were described by Robyr et al. (2002) [19]. The threshold for changes in acetylation state employed in our analyses was arbitrarily set at 1.5-fold based on information in Robyr et al. (2002) [19] indicating that changes above 1.4-fold were not observed in related control experiments.

### Pyxis analysis of clustering and statistical analysis

Datasets were analyzed by Pyxis [17] using a 2-ORF separation window and a maximum p value setting of 0.05. Genes within Pyxis clusters were identified by an in-house Perl-based program that listed all genes between the nucleotide positions for the left end of the first gene in each cluster and the right end of the last gene in each cluster. The probability that observed overlaps between different datasets would occur by chance was calculated using the hypergeometric cumulative distribution function in the Statistics Toolbox of MATLAB [45]. Representation factor is the ratio of the observed number of genes in an overlap compared to the expected number based on the null hypothesis that overlapping datasets arise by chance. Presumably due to rounding off, at low probabilities (<10^-10^), calculation of p varies slightly depending on the order in which values are entered into the MATLAB program.

Genes that map within 1 kilobase (kb) of an ORC-binding locus were identified by in-house built Perl-based programs, which determined whether the starting or ending nucleotide coordinates for genes were found between 1 kb to the left and 1 kb to the right of the coordinates for DNA loci that contain potential ORC binding sites described by [18]. The Monte Carlo method was used to simulate the number of genes that similarly map within 1 kb of an ORC-binding locus based on the assumption that datasets would not exhibit a bias toward genes that map near ORC binding sites. Simulations were performed using in-house built Perl-based programs that randomly selected from a master list of 7104 genes the same number of genes as in experimental datasets and then determined the number of these genes that mapped within 1 kb of a potential ORC-binding locus. This process was iterated 10,000 times and the mean and standard deviation calculated. The number of simulated values greater than or equal to the observed value (or, in the case of under-representation, the number of simulated values less than the observed value) was employed to calculate the P value. The minimum p value for 10,000 simulations is 10^-4^. The master list of genes employed in these simulations included verified ORFS, uncharacterized ORFS, long terminal repeats, transposable element genes and retrotransposons and was downloaded from the Saccharomyces Genome Database [46] on 6/04). All programs are available upon request.

## Authors' contributions

DTB performed Pyxis analyses and all simulations and other statistical analyses, developed software for analyzing Pyxis output and other data and contributed to the preparation of the manuscript. LR provided microarray data in a form suitable for analysis by software and contributed to the interpretation of findings. JW and PL developed software and generated gene maps at early stages of this project. HP developed the Pyxis program and assisted with its implementation. MB contributed to the interpretation of findings. WCB also performed Pyxis analyses, directed this project and contributed to interpretation of findings, as well as preparation of the manuscript.

## Supplementary Material

Additional File 1 "Table S1; Non-random distribution of stress-related genes in relation to ORC binding sites". Summary of distribution of genes in various datasets in relation to ORC binding sites and statistical analysis of the differences between these various datasets Click here for file

Additional File 2 "Table S2; Statistical analysis of overlapping datasets". Summary of statistical analysis of various overlapping datasets Click here for file

Additional File 3 "Chromosome maps of genes induced by starvation or by inactivation of silencing proteins". Comparison between maps of genes and gene clusters detected by Pyxis in datasets of genes induced by starvation or by inactivation of proteins required for chromatin repression of transcription. Click here for file

Additional File 4 "Chromosome maps of genes repressed by starvation or by inactivation of silencing proteins". Comparison between maps of genes and gene clusters detected by Pyxis in datasets of genes repressed by starvation and by inactivation of proteins required for chromatin repression of transcription. Click here for file

Additional File 5 "Table S3; Summary of overlap between various datasets and the dataset of ORFS enriched for acetylated histone H3 in *hda1*Δ compared to wild type cells". Summary of overlap between various datasets and the dataset of ORFS enriched for acetylated histone H3 in *hda1*Δ compared to wild type cells Click here for file

Additional File 6 "Table S4; Summary of overlap between various datasets and the dataset of intergenic regions enriched for acetylated histone H3 in *hda1*Δ compared to wild type cells". Summary of overlap between various datasets and the dataset of intergenic regions enriched for acetylated histone H3 in *hda1*Δ compared to wild type cells Click here for file

Additional File 7 "Comparison between maps of genes and gene clusters detected by Pyxis in datasets of genes induced by starvation or the dataset of genes that confer resistance to stresses". Comparison between maps of genes and gene clusters detected by Pyxis in the Gasch et al. (2000) dataset of genes induced by starvation or the dataset of 974 genes that confer resistance to a variety of stresses identified by decreased viability associated with deletion of these genes Click here for file
