# Supplementary material for: Non-random clustering of stress-related genes during evolution of the S. cerevisiae genome
Source: BMC Evol Biol. 2006 Jul 21;6:58. doi: 10.1186/1471-2148-6-58 (PMC1550265; doi:10.1186/1471-2148-6-58)
Supplement: Additional File 1 — "Table S1; Non-random distribution of stress-related genes in relation to ORC binding sites". Summary of distribution of genes in various datasets in relation to ORC binding sites and statistical analysis of the differences between these various datasets [file 1471-2148-6-58-S1.pdf]

Table S1
Non-random distribution of stress-related genes in relation to ORC binding sites

Dataset	Number of genes in dataset	Number of genes in dataset within 1 kb ORC binding site	% total genes in dataset	Average number of simulated genes within 1 kb ORC binding site [s.d.]	% total dataset simulated genes that are within 1 kb ORC binding site (P)
all genes*	7104	1164	16.4%	1164 [9.2]	16.4% (1)
starvation induced genes	2213	459	20.7%	363 [14.4]	16.4% ($1 X10^{-4}$)
starvation-repressed genes	2020	244	12.1%	331 [14.0]	16.4% ($1 X10^{-4}$)
potentially silenced genes	2111	431	20.4%	346 [14.1]	16.4% ($1 X10^{-4}$)
starvation induced clusters	1404	323	23.1%	230 [12.3]	16.4% ($1 X10^{-4}$)
starvation repressed clusters	1416	184	13%	232 [12.5]	16.4% ($2 X10^{-4}$)
potentially silenced gene clusters	1201	289	24.1%	197 [11.6]	16.4% ($1 X10^{-4}$)
Genes essential for growth	1106	130	11.8%	181 [11.2]	16.4% ($1 X10^{-4}$)
essential gene clusters	714	91	12.7	117 [9.3]	16.4% ($3 X10^{-3}$)
H_2O_2-induced genes	1379	255	18.5	226 [12.5]	16.4% ($1 X10^{-2}$)
H_2O_2-repressed genes	1536	235	15.3%	252 [12.9]	16.4% ($1 X10^{-1}$)
ESR-induced genes	282	63	22.3%	46 [6.1]	16.4% ($5 X10^{-3}$)
ESR-repressed genes	585	75	12.8%	96 [8.6]	16.4% ($9 X10^{-3}$)
H_2O_2-resistance genes	123	15	12.2%	20 [4.0]	16.4% ($1 X10^{-1}$)
oxidative stress resistant	685	87	12.7%	112 [9.1]	16.4% ($3 X10^{-3}$)
HU-induced genes	116	24	20.5%	19 [4.0]	16.4% ($1 X10^{-1}$)
HU-repressed genes	71	7	9.8%	12 [3.2]	16.4% ($9 X10^{-2}$)

HU-resistance genes	136	13	9.6%	22 [4.2]	16.4% (2 X10^{-2})
MMS-induced genes	452	93	20.6%	74 [7.6]	16.4% (9 X 10^{-3})
MMS-repressed genes	217	33	15.2%	35 [5.3].	16.4% (4 X10^{-1})
MMS-resistance genes	103	11	10.7%	17 [3.7]	16.4% (7 X10^{-2})
camptothecin-sensitive genes	83	8	9.6%	14 [3.4]	16.4% (6 X10^{-2})
UV-resistance genes	307	34	11.1%	50 [6.4]	16.4% (5 X10^{-3})
slowly evolving paralogues	115	15	13.0%	19 [4.0]	16.4% (2 X10^{-1})
rapidly evolving paralogues	115	26	22.6%	19 [3.9]	16.4% (5 X10^{-2})

Table S1. Non-random distribution of stress-related genes in relation to ORC binding sites. Genes that map within 1 kilobase (kb) of an ORC-binding locus were identified by in-house built Perl-based programs, which determined whether the starting or ending nucleotide coordinates for genes were found between 1 kb to the left and 1 kb to the right of the coordinates for DNA loci that contain potential ORC binding sites described by [14]. The Monte Carlo method was used to simulate the number of genes that similarly map within 1 kb of an ORC-binding locus based on the assumption that datasets would not exhibit a bias toward genes that map near ORC binding sites. Simulations were performed using in-house built Perl-based programs that randomly selected from a master list of 7104 genes (including transposon-related genes) the same number of genes as in experimental datasets and then determined the number of these genes that mapped within 1 kb of a potential ORC-binding locus. This process was iterated 10,000 times and the mean and standard deviation calculated. The number of simulated values greater than or equal to the observed value (or, in the case of under-representation, the number of simulated values less than the observed value) was employed to calculate the P value. "Starvation-induced" and "starvation-repressed" datasets correspond to genes induced or repressed 2-fold or more between 8 hours and 28 days of medium depletion (*1*). "Starvation-induced clusters" and "starvation-repressed clusters" datasets correspond to all genes found in statistically significant ($p < 0.05$) physical clusters by the Pyxis program in these starvation-induced or starvation-repressed datasets. See Materials and Methods of Burhans et al. for descriptions of other datasets.
