# Supplementary material for: Non-random clustering of stress-related genes during evolution of the S. cerevisiae genome
Source: BMC Evol Biol. 2006 Jul 21;6:58. doi: 10.1186/1471-2148-6-58 (PMC1550265; doi:10.1186/1471-2148-6-58)
Supplement: Additional File 2 — "Table S2; Statistical analysis of overlapping datasets". Summary of statistical analysis of various overlapping datasets [file 1471-2148-6-58-S2.pdf]

Table S2. Statistical analysis of overlapping datasets

Genelist 1	Number genes in genelist 1	Genelist 2	Number genes in genelist 2	Number genes in overlap	Represent-ation factor	P value
**starvation induced genes**	2213	potentially silenced genes	2111	1086	1.4	5.2×10^{-12}
**starvation induced clusters**	1404	*orc2-1* induced (≥ 2-fold)	524	193	1.6	1.6×10^{-11}
**starvation induced clusters**	1404	starvation repressed clusters	1416	32	0.1	4.8×10^{-132}
**starvation induced clusters**	1404	all starvation repressed genes	2020	105	0.2	3.8×10^{-139}
**starvation induced clusters**	1404	potentially silenced genes	2111	644	1.4	1.9×10^{-11}
**starvation induced clusters**	1404	potentially silenced clusters	1139	559	2.2	2.2×10^{-11}
**starvation induced clusters**	1404	essential growth genes	1106	106	0.4	1.5×10^{-35}
**starvation induced clusters**	1404	essential gene clusters	1467	140	0.4	1.8×10^{-49}
**starvation induced clusters**	1404	H_20_2 induced	1379	434	1.4	2.2×10^{-11}
**starvation induced clusters**	1404	H_20_2 repressed	1536	224	0.6	1.4×10^{-20}
**starvation induced clusters**	1404	H_20_2-resistant	123	13	0.5	4.0×10^{-4}
**starvation induced clusters**	1404	oxidative stress resistant	685	95	0.6	7.2×10^{-10}
**starvation induced genes**	2213	HU-induced	116	83	2.0	$< 10^{-20}$
**starvation induced clusters**	1404	HU-induced	116	41	1.6	< 0.001

**starvation induced clusters**	1404	HU-resistant	136	9	0.3	3.8×10^{-7}
**starvation induced clusters**	1404	MMS- induced	452	181	1.8	< 0.001
**starvation repressed clusters**	1416	MMS-induced	452	55	0.5	< 0.001
**starvation induced clusters**	1404	MMS-repressed	217	20	0.4	< 0.001
**starvation repressed clusters**	1416	MMS-repressed	217	87	1.8	< 0.001
**starvation induced clusters**	1404	MMS- resistant	103	6	0.3	3.2×10^{-6}
**starvation induced clusters**	1404	ESR- induced	282	123	2.0	1.9×10^{-11}
**starvation repressed genes**	2020	potentially silenced genes	2111	296	0.4	1.1×10^{-121}
**starvation repressed genes**	2020	essential growth genes	1106	620	1.7	4.2×10^{-12}
**starvation repressed clusters**	1416	ESR-induced	282	14	0.2	< 0.001
**starvation induced clusters**	1404	ESR-repressed	585	14	0.1	< 0.001
**starvation repressed clusters**	1416	ESR-repressed	585	322	2.4	< 0.001
**starvation induced clusters**	1404	camptothecin-resistant	83	7	0.4	5.6×10^{-4}
**starvation induced clusters**	1404	UV resistant	307	30	0.4	< 0.001
**starvation induced genes**	2213	essential growth genes	1106	136	0.3	$< 10^{-20}$
**starvation repressed clusters**	1416	potentially silenced clusters	1139	81	0.3	2.4×10^{-54}

**starvation repressed clusters**	1416	*orc2-1* induced (≥ 2-fold)	524	42	0.4	2.6×10^{-21}
**potentially silenced genes**	2111	essential growth genes	1106	161	0.4	4.2×10^{-58}
**all stress-resistance**	974	potentially silenced	2111	251	0.8	5.7×10^{-10}
**slowly evolving paralogues**	115	potentially silenced	2111	19	0.5	6.8×10^{-6}
**rapidly evolving paralogues**	115	potentially silenced	2111	57	1.5	3.8×10^{-4}
**slowly evolving paralogues**	115	starvation repressed	2020	49	1.3	< 0.03
**rapidly evolving paralogues**	115	starvation repressed clusters	1416	16	0.6	< 0.02
**rapidly evolving paralogues**	115	starvation induced genes	2213	61	1.5	<0.001

Table S1. Statistical analysis of overlapping datasets. The *orc2-1* datasets corresponds to genes induced by the *orc2-1* mutation six hours after shifting to the non-permissive temperature of 35°C[1]. "Starvation-induced" and "starvation-repressed" datasets correspond to genes induced or repressed 2-fold or more between 8 hours and 28 days of medium depletion[2]. "Starvation-induced clusters" and "starvation-repressed clusters" datasets correspond to all genes found in statistically significant ($p < 0.05$) physical clusters by the Pyxis program in the starvation-induced or starvation-repressed datasets (this study – the Pyxis program is described by Chang et al. (2004)[3]. Genes in these clusters were identified by an in-house Perl-based program that listed in the output all genes between the nucleotide coordinates for the left end of the first gene and the right end of the last gene in each gene cluster identified by the Pyxis program (the coordinates contained in the output of the Pyxis program are not correct and were ignored). The "potentially silenced" dataset corresponds to genes induced 2-fold or more after 6 hour depletion of histone H4 or by deletion of the *SIR2*, *SIR3*, or *SIR4* genes [4], deletion of the *TUP1* gene[5], or by the *orc2-1* mutation[1]. "Essential growth gene" dataset corresponds to genes required for growth in rich medium[6]. "HU-induced" dataset corresponds to genes induced by 1 hour treatment of cells with HU (hydroxyurea)[7]. "MMS-induced" corresponds to all genes induced 3-fold or more by MMS (methyl methanesulfonate")[8]. "HU-resistant", "MMS-resistant", "camptothecin-resistant" and "cycloheximide-resistant" datasets correspond to genes that, when deleted, confer sensitivity to hydroxyurea, MMS, camptothecin or cycloheximide respectively[9]. "H_2O_2-resistant" dataset corresponds to genes that, when deleted, confer sensitivity to H_2O_2[10] and "H_2O_2-induced" dataset corresponds to genes induced between 5'-120' of H_2O_2 treatment[2] P values (probability that overlaps would occur by chance) were calculated using the hypergeometric cumulative distribution function in the Statistics Toolbox of Matlab (http://www.mathworks.com). Representation factor is the ratio of the observed number of genes in the overlap compared to the expected number based on the null hypothesis that overlapping datasets arise by chance.

References:

1. L Ramachandran, P Liang, J Wang, M Weinberger, DT Burhans, S Wissing, S Jarolim, B Suter, F Madeo, M Breitenbach and WC Burhans: Evidence for ORC-dependent repression of budding yeast genes induced by starvation and other stresses. *FEMS Yeast Research* 2006, **6:**763-776.
2. Gasch, A. P. et al. Genomic expression programs in the response of yeast cells to environmental changes. *Mol Biol Cell* **11**, 4241-57 (2000).
3. Chang, C. F., Wai, K. M. & Patterton, H. G. Calculating the statistical significance of physical clusters of co-regulated genes in the genome: the role of chromatin in domain-wide gene regulation. *Nucleic Acids Res* **32**, 1798-807 (2004).
4. Wyrick, J. J. et al. Chromosomal landscape of nucleosome-dependent gene expression and silencing in yeast. *Nature* **402**, 418-21 (1999).
5. DeRisi, J. L., Iyer, V. R. & Brown, P. O. Exploring the metabolic and genetic control of gene expression on a genomic scale. *Science* **278**, 680-6 (1997).
6. Winzeler, E. A. et al. Functional characterization of the S. cerevisiae genome by gene deletion and parallel analysis. *Science* **285**, 901-6. (1999).
7. Ostapenko, D. & Solomon, M. J. Budding yeast CTDK-I is required for DNA damage-induced transcription. *Eukaryot Cell* **2**, 274-83 (2003).
8. Fry, R. C., Sambandan, T. G. & Rha, C. DNA damage and stress transcripts in Saccharomyces cerevisiae mutant sgs1. *Mech Ageing Dev* **124**, 839-46 (2003).
9. Parsons, A. B. et al. Integration of chemical-genetic and genetic interaction data links bioactive compounds to cellular target pathways. *Nat Biotechnol* **22**, 62-9 (2004).
10. Thorpe, G. W., Fong, C. S., Alic, N., Higgins, V. J. & Dawes, I. W. Cells have distinct mechanisms to maintain protection against different reactive oxygen species: oxidative-stress-response genes. *Proc Natl Acad Sci U S A* **101**, 6564-9 (2004).
