# Supplementary figures and images for: Non-random clustering of stress-related genes during evolution of the S. cerevisiae genome
Source: BMC Evol Biol. 2006 Jul 21;6:58. doi: 10.1186/1471-2148-6-58 (PMC1550265; doi:10.1186/1471-2148-6-58)

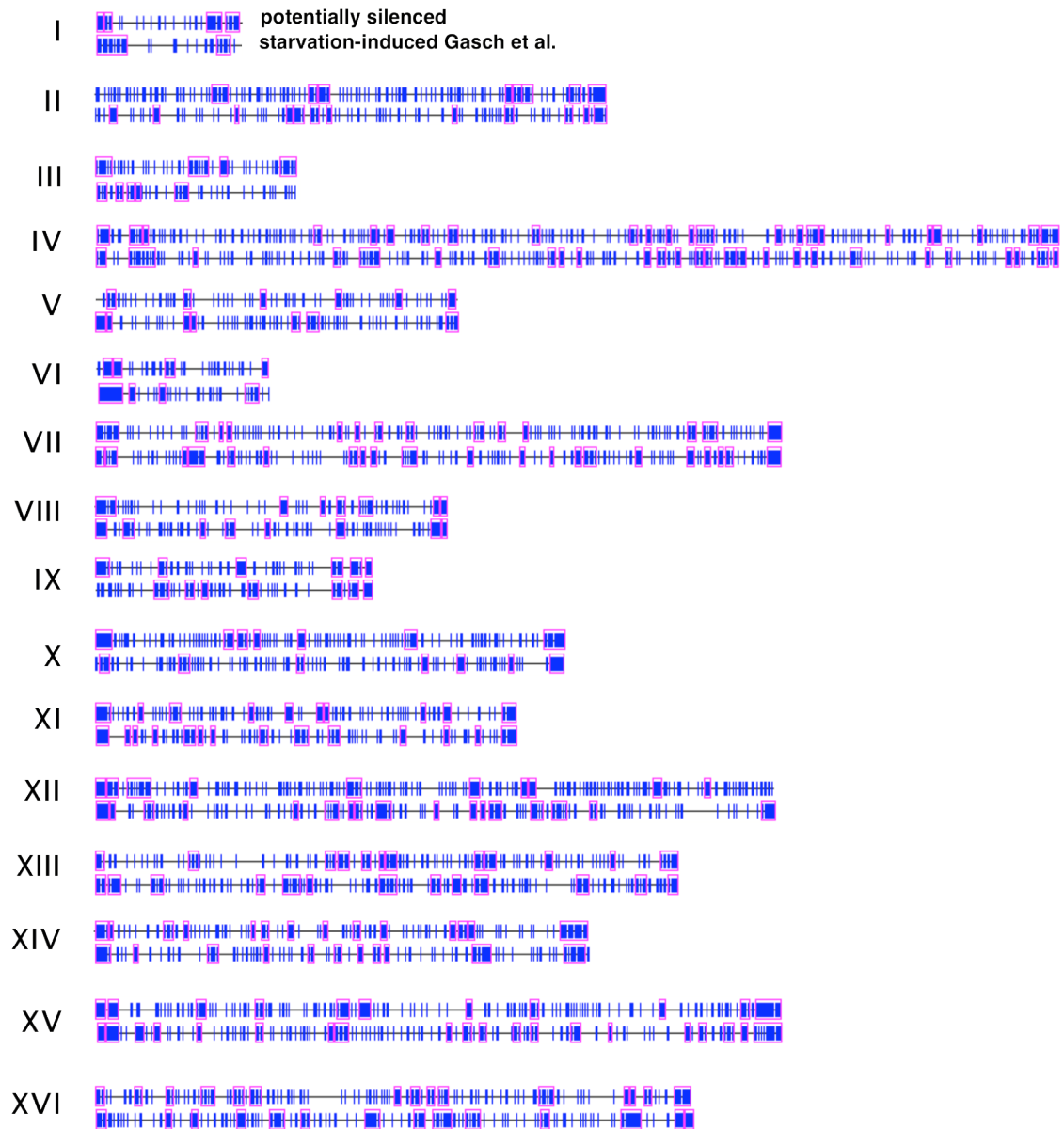

Supplement: Additional File 3 — "Chromosome maps of genes induced by starvation or by inactivation of silencing proteins". Comparison between maps of genes and gene clusters detected by Pyxis in datasets of genes induced by starvation or by inactivation of proteins required for chromatin repression of transcription. [file 1471-2148-6-58-S3.pdf]

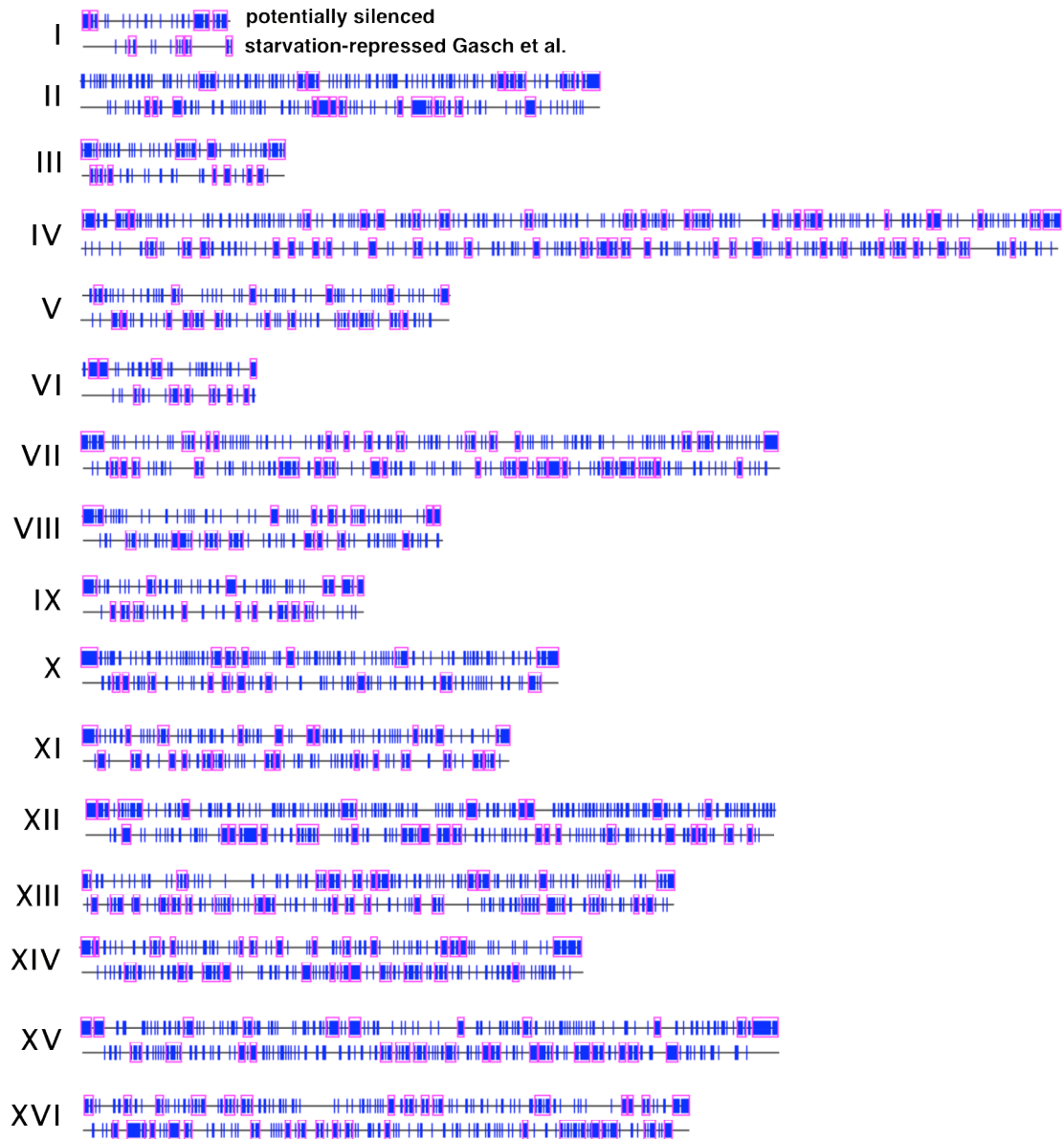

Supplement: Additional File 4 — "Chromosome maps of genes repressed by starvation or by inactivation of silencing proteins". Comparison between maps of genes and gene clusters detected by Pyxis in datasets of genes repressed by starvation and by inactivation of proteins required for chromatin repression of transcription. [file 1471-2148-6-58-S4.pdf]

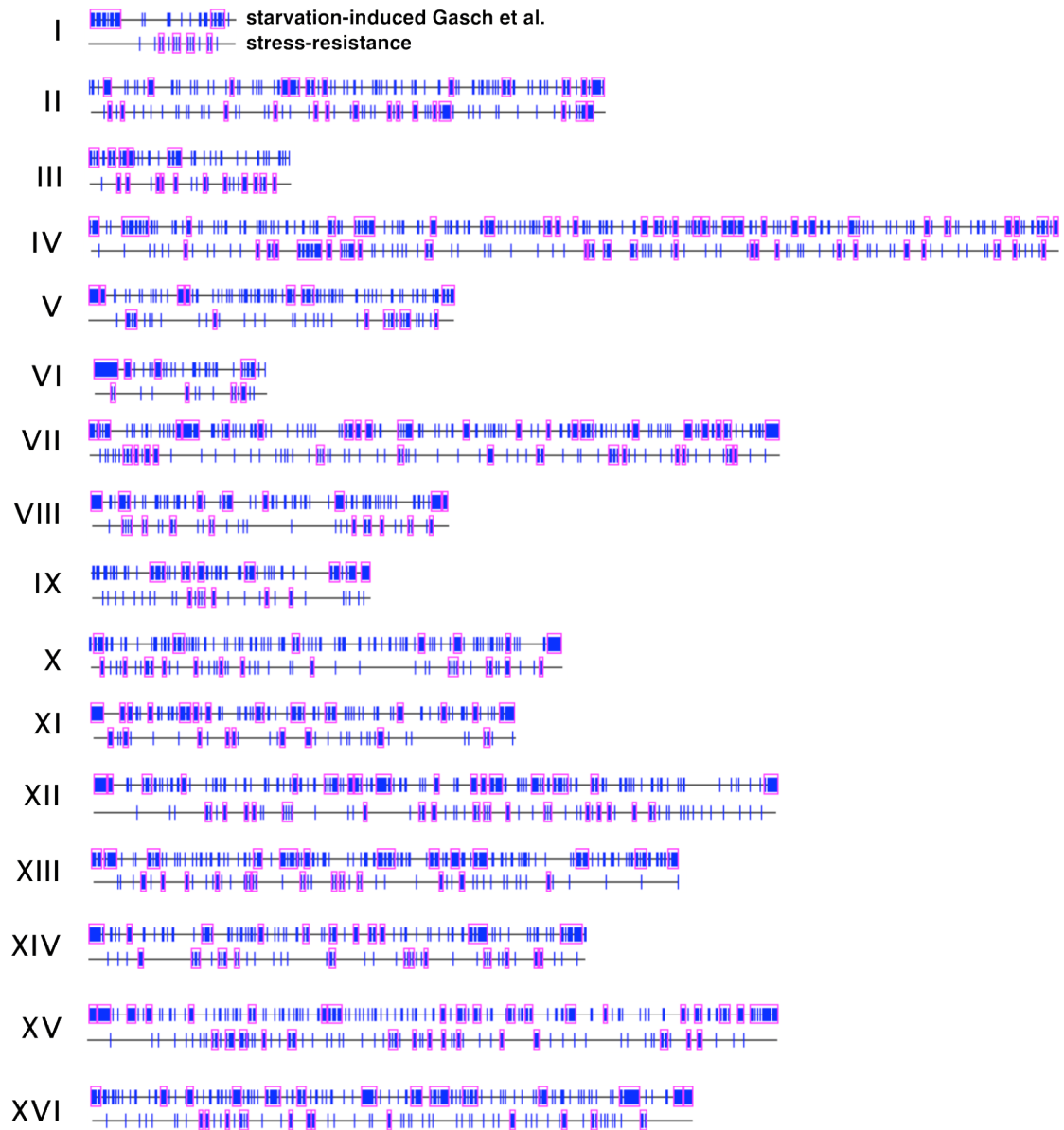

Supplement: Additional File 7 — "Comparison between maps of genes and gene clusters detected by Pyxis in datasets of genes induced by starvation or the dataset of genes that confer resistance to stresses". Comparison between maps of genes and gene clusters detected by Pyxis in the Gasch et al. (2000) dataset of genes induced by starvation or the dataset of 974 genes that confer resistance to a variety of stresses identified by decreased viability associated with deletion of these genes [file 1471-2148-6-58-S7.pdf]
