# Supplementary material for: Non-random clustering of stress-related genes during evolution of the S. cerevisiae genome
Source: BMC Evol Biol. 2006 Jul 21;6:58. doi: 10.1186/1471-2148-6-58 (PMC1550265; doi:10.1186/1471-2148-6-58)
Supplement: Additional File 5 — "Table S3; Summary of overlap between various datasets and the dataset of ORFS enriched for acetylated histone H3 in hda1Δ compared to wild type cells". Summary of overlap between various datasets and the dataset of ORFS enriched for acetylated histone H3 in hda1Δ compared to wild type cells [file 1471-2148-6-58-S5.pdf]

Supplementary Table S3

Frequency with which genes induced or repressed by medium depletion starvation or that are essential for growth are hyperacetylated in *hda1Δ* compared to wild type cells

Dataset	# Genes in dataset	# ORFs acetylated at H3 K18 ≥ 1.5-fold in *hda1Δ* compared to wild type cells	% genes in dataset increased in acetylation at H3 K18	P	representation factor
**All ORFs***	**6070**	**2418 (**	**39.8%**	**1.0**	**1.0**
**ORFs induced by starvation[1]**	**2117**	**1147**	**54.1%**	**> 0.001**	**1.4**
**ORFs in all starvation-induced clusters**	**1162**	**664**	**57.1%**	**> 0.001**	**1.4**
**ORFs in starvation-induced clusters within 50 kb of telomeres**	**305**	**179**	**58.6%**	**> 0.001**	**1.5**
**ORFs within 50 kb telomeres, but not in starvation-induced clusters**	**388**	**166**	**42.8%**	**0.128**	**1.1**
**ORFs in internal starvation-induced clusters (> 50 kb of telomeres)**	**873**	**493**	**56.5%**	**> 0.001**	**1.4**
**ORFs repressed by starvation[1]**	**1976**	**492**	**24.9%**	**> 0.001**	**0.6**
**ORFs in starvation-repressed clusters**	**1262**	**322**	**25.5%**	**> 0.001**	**0.6**
**ORFs not in starvation-induced or starvation-repressed clusters and > 50 kb from telomeres**	**3265**	**1274**	**39.0%**	**0.166**	**1.0**
**ORFs encoding essential proteins**	**1106**	**282**	**25.5%**	**> 0.001**	**0.6**

* ~6070 ORFs represented in “ORF” microarrays employed in the study by Robyr et al. (2002) [1]. [1]induced or repressed by medium depletion starvation according to Gasch et al. (2000) [2]. These and other starvation-related datasets were stripped of transposon-related sequences before calculating acetylation frequency based on the ORF acetylation data of Robyr et al., which did not contain these sequences.

P values (probability that overlaps would occur by chance) were calculated using the hypergeometric cumulativedistribution function in the Statistics Toolbox of Matlab (http://www.mathworks.com). Representation factor is the ratio of the observed number of genes in the overlap compared to the expected number based on the null hypothesis that overlapping datasets arise by chance.

1. D Robyr, Y Suka, I Xenarios, SK Kurdistani, A Wang, N Suka, M Grunstein: **Microarray deacetylation maps determine genome-wide functions for yeast histone deacetylases**. *Cell* 2002, **109**:437-46.
2. AP Gasch, PT Spellman, CM Kao, O Carmel-Harel, MB Eisen, G Storz, D Botstein, PO Brown: **Genomic expression programs in the response of yeast cells to environmental changes**. *Mol Biol Cell* 2000, **11**:4241-57.
