# Supplementary material for: Non-random clustering of stress-related genes during evolution of the S. cerevisiae genome
Source: BMC Evol Biol. 2006 Jul 21;6:58. doi: 10.1186/1471-2148-6-58 (PMC1550265; doi:10.1186/1471-2148-6-58)
Supplement: Additional File 6 — "Table S4; Summary of overlap between various datasets and the dataset of intergenic regions enriched for acetylated histone H3 in hda1Δ compared to wild type cells". Summary of overlap between various datasets and the dataset of intergenic regions enriched for acetylated histone H3 in hda1Δ compared to wild type cells [file 1471-2148-6-58-S6.pdf]

Supplementary Table S4

Genes induced or repressed by medium depletion starvation and their relationship to intergenic regions hyperacetylated in *hda1Δ* compared to wild type cells

Dataset	% genes associated with intergenic regions acetylated at H3K18 ≥ 1.5-fold in *hda1Δ* compared to wild type cells
**All genes***	**27.6%**
**genes in starvation-induced clusters**[1]	**38.4%**
**genes in starvation-induced clusters within 50 kb of telomeres**	**41.0%**
**genes in internal starvation-induced clusters (> 50 kb from telomeres)**	**38.0%**
**genes in starvation-repressed clusters**[1]	**16.8%**
**genes not in starvation-induced or starvation-repressed clusters and > 50 kb from telomeres**	**26.7%**
**genes essential for growth**	**22.6%**

* "All genes" includes ORFs and transposon-related genes induced or repressed by medium depletion starvation according to Gasch et al. (2000) [2]
Intergenic acetylation data are from Robyr et al. (2002) [1].

1. D Robyr, Y Suka, I Xenarios, SK Kurdistani, A Wang, N Suka, M Grunstein: **Microarray deacetylation maps determine genome-wide functions for yeast histone deacetylases**. *Cell* 2002, **109**:437-46.
2. AP Gasch, PT Spellman, CM Kao, O Carmel-Harel, MB Eisen, G Storz, D Botstein, PO Brown: **Genomic expression programs in the response of yeast cells to environmental changes**. *Mol Biol Cell* 2000, **11**:4241-57.
